# Vitamin D Supplementation: Effect on Cytokine Profile in Multiple Sclerosis

**DOI:** 10.3390/jcm13030835

**Published:** 2024-02-01

**Authors:** Maddalena Sparaco, Simona Bonavita

**Affiliations:** Department of Advanced Medical and Surgical Sciences, University of Campania Luigi Vanvitelli, 80138 Naples, Italy; maddalena.sparaco@policliniconapoli.it

**Keywords:** vitamin D, multiple sclerosis, cytokines

## Abstract

Vitamin D is known for its role in modulating calcium and phosphate homeostasis and is implicated both in bone mineralization and immune system regulation. The immune-modulatory role of vitamin D and its impact on multiple sclerosis (MS) courses are still debated. The aim of this review was to check the effect of vitamin D supplementation on cytokine profile regulation in people with MS. A significant increase in serum concentrations of interleukin (IL)-10 and Transforming growth factor (TGF)-β1 after vitamin D supplementation was demonstrated in most studies, with some of them reporting a reduction in disability scores after vitamin D supplementation and an inverse correlation between IL-10 levels and disability. The effect of vitamin D on the serum levels of IL-17 and IL-6 was controversial; different results across studies could be explained by a variability in the treatment duration, route, and frequency of administration, as well as the dosage of vitamin D supplementation, responses to vitamin D treatment and the serum levels reached with supplementation, including the methods used for cytokine analysis and the different cell types investigated, the MS phenotype, the disease phase (active vs. non-active) and duration, and concomitant treatment with disease-modifying therapies. Nevertheless, the significant increase in the serum concentrations of IL-10 and TGF-β1, demonstrated in most studies, suggests an anti-inflammatory effect of vitamin D supplementation.

## 1. Introduction

Multiple sclerosis (MS) is a chronic autoimmune disease that predominantly affects young adults, especially women [1]. Since Charcot’s first descriptions, many steps forward have been taken in the understanding and management of MS that led to a better definition of etiopathology, an early diagnosis, a prediction of the clinical course, and a tailored treatment [2].

MS is not confined to the white matter, but it also involves the gray matter. It is a neuroinflammatory and neurodegenerative disease [2]. The etiology of MS is considered multifactorial, with a combination of genetic susceptibility and environmental risk factors (including infection, in particular, the Epstein–Barr virus (EBV), as well as vitamin D low serum levels, ultraviolet-B light (UV-B) exposure, obesity, and smoking) [2,3,4,5,6]. The MS onset and course may be influenced by sex hormone changes, considering the onset of MS in post-puberty age, the impact of pregnancy and puerperium on the MS course, and clinical changes during climacteric, as well as the debated prognostic role of male sex [6]. Despite MS historically being described as a T-cell-mediated autoimmune disease, the role of B cells has recently been emphasized, leading to the advent of B-cell-targeted therapies [2]. The number and efficacy of disease-modifying therapies (DMTs) have increased, evolving from the first immunomodulatory drugs (such as interferon beta and glatiramer acetate) to more recent DMTs, i.e., the sphingosine-1-phosphate modulators, NFκB downregulator, dihydro-orotate dehydrogenase inhibitor, deoxyadenosine analog, a monoclonal antibody directed against the α4β1 integrin or the CD-20 + B cells, or the cell surface CD52 antigen [2].

The role of prognostic factors, the identification of suitable biomarkers, and the impact of comorbidities on disease progression are widely discussed topics in the context of MS. One of the debated issues pertains to the role of vitamin D in the onset and course of MS.

Vitamin D is a steroid hormone originating from two sources: endogenous, by human skin, and exogenous, through dietary intake or supplementation [2]. Vitamin D is mainly synthesized by human skin after exposure to UV-B (in the range of 290–315 nm). It then undergoes hydroxylation, primarily in the liver, to be transformed into Calcifediol (25-OH D3) and, subsequently, in the kidney to generate Calcitriol (1,25-OH2 D3) [7]. 1,25-OH2D3 binds to the intracellular vitamin D receptor (VDR), which heterodimerizes with retinoid-X-receptor (RXR). In the nucleus, the 1,25-OH2D3/VDR/RXR complex binds to the vitamin D-responsive elements in the DNA and modulates the expression of target genes [7].

Vitamin D is known for its role in modulating calcium and phosphate homeostasis, and it is also implicated both in bone mineralization and in the regulation of the cardiovascular system, glucose/lipid metabolism, and the immune system.

In particular, vitamin D is implicated in the regulation of both innate and acquired immunity. The VDR and the vitamin D-activating enzyme have been reported in various types of immune cells, including monocytes, macrophages, dendritic cells, and T and B lymphocytes [8].

Vitamin D inhibits the differentiation and maturation of dendritic cells (modulating the expression of costimulatory molecules, such as CD40, CD80, and CD86 and class II MHC molecules), limiting the activation and reducing the proliferation of alloreactive T cells and promoting the spontaneous apoptosis of mature dendritic cells [9].

Direct effects of vitamin D on T lymphocytes have also been reported: it inhibits Th1 cells and enhances the Th2 cell phenotype, favoring the expression of the transcription factors, such as c-maf and GATA-3, correlated with the increased production of Th2 cytokines [10].

Furthermore, vitamin D can confer an anti-inflammatory profile to EBV-specific CD8+ T cells independently from other immune cells, including CD4+ T cells, dendritic cells, and B lymphocytes [11]. Considering the crucial role of EBV infection in MS onset, this finding is highly relevant in the context of MS: indeed, it has been demonstrated that there is an inverse correlation between vitamin D serum concentrations and EBV load in people with MS (pwMS) [4].

Also, the effect of vitamin D on B-cell function has been reported: vitamin D plays a role in the maintenance of B-cell homeostasis by inhibiting differentiation (and consequently, the generation of memory B cells and plasma cells and immunoglobulin production) and regulating the proliferation of activated B cells [12].

The impact of vitamin D on regulating both innate and acquired immunity suggests its possible immunomodulatory role in autoimmune diseases such as MS.

An association between serum vitamin D levels and MS was extensively investigated, demonstrating its potential influence on both the onset and disease course [5,13,14,15,16,17,18].

The high prevalence of MS in high latitude areas might support a possible relation between MS and vitamin D serum levels associated with sun exposure [9,10]. Living in areas with high UV-B exposition at ages 5 to 15 years, as well as during the period from 5 to 15 years before MS onset, was associated with a reduced risk of developing MS compared to living in low UV-B areas [10]. Furthermore, vitamin D serum levels, measured at the time of MS diagnosis between June and September, were lower in pwMS than in the controls [14].

The vitamin D serum concentration and treatment with vitamin D supplementation in add-on DMT might influence the clinical and radiological activity of MS [15,16,17,18]. An effect in reducing the relapse rate, disease progression, the rate of new/enlarging T2 lesions, the number of T1-enhancing lesions, and brain volume loss have been reported in several studies, including randomized, double-blind, placebo-controlled trials [15,16,17,18]. Furthermore, patients with secondary progressive (SP) MS have lower vitamin D levels when compared to relapsing-remitting (RR) MS, and an association between low vitamin D levels and early conversion to SPMS has been demonstrated [19]. Additionally, in people with clinically isolated syndrome (CIS), higher vitamin D levels predict reduced MS activity and a slower rate of progression [15].

The role of vitamin D has also been emphasized, in particular, in life conditions of women with MS, such as the puerperium and during artificial reproductive technology (ART) treatment, which is characterized by an increased risk of relapse [20,21,22,23,24]. Although the association between vitamin D levels and the risk of post-partum relapse has not always been confirmed [21], vitamin D supplementation could reduce the relapse rate during pregnancy and within 6 months after delivery [22]. Moreover, vitamin D deficiency has been associated with worse ART outcomes [23,24], and ART failure could increase the risk of relapse [25].

The mechanism by which vitamin D promotes this effect is still under investigation. In the murine model of experimental autoimmune encephalomyelitis (EAE), treatment with 1,25-OH2 D3 significantly decreased the production/expression of pro-inflammatory cytokines (such as interferon (IFN)-γ and interleukin (IL)-17A) and increased anti-inflammatory ones (i.e., IL-4 and IL-10), also promoting oligodendrocyte differentiation and maturation [26].

Therefore, the debate on the role of vitamin D supplementation as an immune modulator and its impact on the MS course is still open. The aim of this review is to investigate the effect of vitamin D supplementation on serum cytokine profile regulation in pwMS and the consequent effect on MS.

## 2. Literature Search Methodology

The literature search was conducted using PubMed/Medline and Scopus as electronic databases. The studies were identified using a combination of the following medical subject heading (MeSH) terms: “Multiple Sclerosis” and “vitamin D” and “Cytokine”, “interleukine”, “IL”, “transforming growth factor”, “TGF”, “interferon-gamma”, “INF-gamma”. Restrictions to English articles were applied. No date restrictions were used.

## 3. Results

### 3.1. Study Selection

**Figure 1:**
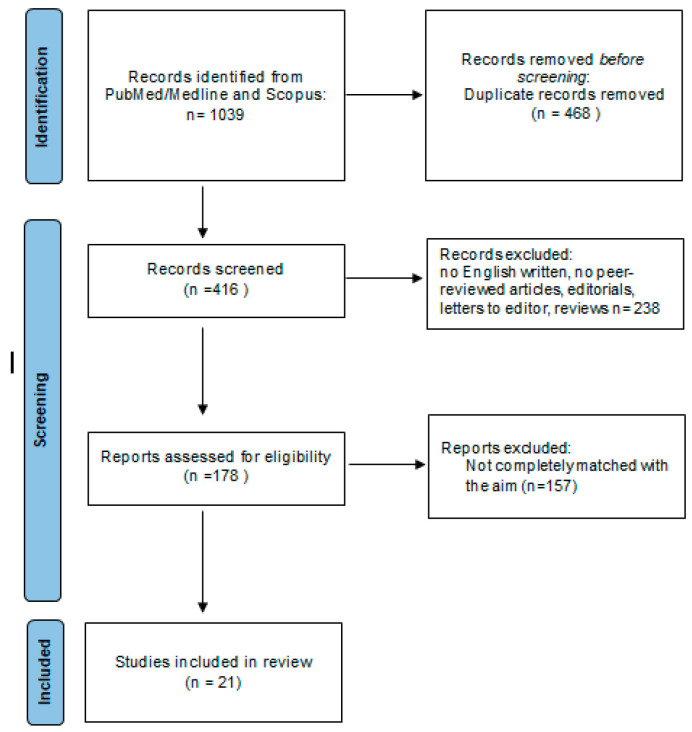
PRISMA flow diagram that includes searches of PubMed/Medline and Scopus.

A total of 1039 results were retrieved. Duplications were removed using Endnote (online version), and manually. Only English-written peer-reviewed studies were considered for inclusion. Finally, 21 full research articles were included, considering only the studies focusing on the effect of vitamin D supplementation on the serum cytokine profile regulation in pwMS. The workflow of the literature selection is described in the PRISMA flowchart (Figure 1).

### 3.2. Vitamin D effect on Cytokine Profile in MS

- **IL-10**

Interleukin (IL)-10 is an anti-inflammatory cytokine with an important role as a regulator of the immune response. Although IL-10 was initially described as secreted by Th2 cell clones, it is known to be secreted by several cells of the innate and acquired immune system, including macrophages, monocytes, dendritic cells, mast cells, eosinophils, neutrophils, natural killer cells, and T and B cells [27].

IL-10 acts on antigen-presenting cells, inhibiting the release of pro-inflammatory mediators (TNF-α, IL-1β, IL-6, IL-8, G-CSF, and GM-CSF) and reducing the expression of MHC II and co-stimulating and adhesion (CD86 and CD54) molecules. Moreover, IL-10 inhibits both the differentiation and proliferation of CD4+ T cells and cytokine production by CD4+ T cells [27].

In pwMS, peripheral blood B cells secrete less IL-10 compared with healthy controls (HCs) [28,29], and T cells present resistance to IL-10-mediated immunosuppression [28]. Furthermore, pwMS with high IL-10 production (measured in stimulated lymphocytes from peripheral blood) had significantly lower disability scores and a lower T2 lesion load [30]. In people with CIS, low serum IL-10 levels were associated with a higher risk and shorter interval to second events and correlated with a significantly higher white blood cell count in cerebrospinal fluid (CSF), number of T2 lesions, and gadolinium-enhancing lesions at the baseline MRI [31].

Studies on healthy people have demonstrated the effect of vitamin D in the regulation of IL-10 production. By adding 1,25-OH2 D3 to a culture of peripheral blood mononuclear cells (PBMCs), Penna et al. found an enhanced IL-10 production and inhibited IL-12 secretion [9]. The variation in the levels of these cytokines is linked to the inhibition of dendritic cell maturation and the promotion of the spontaneous apoptosis of mature dendritic cells [9]. Correale et al. evaluated the direct effects of 1,25-OH2 D3 on ex vivo freshly isolated CD4+ T cells and myelin-peptide-specific T-cell lines, demonstrating the enhanced development of IL-10-producing cells by vitamin D [32].

The effect of vitamin D on IL-10 in pwMS has been largely studied, measuring serum IL-10 levels after vitamin D supplementation (see Table 1 and Table 2).

Walawska-Hrycek et al. observed a significant increase in serum concentrations of IL-10 after 12 months of vitamin D treatment (with the administered dose depending on the degree of deficiency, ranging from 500 to 1000 UI/die for 12 months) [33]. This study confirmed the results of Smolders et al., who found an increased proportion of IL-10+ CD4+ T cells after supplementation with 20,000 IU/die of vitamin D for 12 weeks [34]. Also, other studies confirmed the increased expression of IL-10 after treatment with 50,000 IU of vitamin D (every week for 8 weeks, or every 5 days for 3 months) [35,36,37,38] or 300,000 IU of vitamin D intramuscular injection every month for 6 months [39]. Moreover, Shirvani-Farsani et al. also demonstrated a positive association between serum concentrations of 25-OH D3 and IL-10 levels [36].

However, these data were not confirmed by Åivo et al., Mrad et al., Golan et al., and Rolf et al. [40,41,42,48]. In these studies, no change in the IL-10 serum levels was found after vitamin D treatment (with 20,000 IU/week for 12 months [42], 10,000 IU daily for 12 weeks [41], 800 IU daily or 800 IU daily + 75,000 UI every 3 weeks for 12 months [40], or 7000 IU daily in the first 4 weeks, followed by 14,000 IU daily up to week 48 [48]).

- **IL-17**

IL-17 is produced by a new class of CD4+ T cells (Th17), γδ T cells, and innate lymphoid cells. Since the first description of Th17 cells in 2005, IL-17 has been widely studied, leading to the identification of the Th17 cells and IL-17’s role in the inflammatory processes of MS [49]. The presence of Th17 cells and high levels of IL-17 were detected in plasma and CSF in the active phase of MS [49,50,51]. Moreover, IL-17 promotes the production of pro-inflammatory cytokines and chemokines (including IL-6), favoring the differentiation of naïve T cells into the Th17 lineage and the migration of Th17 cells to the site of inflammation [50].

The number of Th17 cells is significantly higher in the CSF of RR pwMS during relapse compared to the remission phase or other non-inflammatory neurological diseases [51].

Correale et al. evaluated the direct effects of 1,25-OH2 D3 on freshly isolated CD4+ T cells and myelin-peptide-specific T-cell lines, demonstrating fewer IL-6 and IL-17 producing cells and inhibited IL-17 production by activated vitamin D [32].

The effect of vitamin D on the IL-17 serum level in pwMS was extensively investigated, with controversial results (see Table 1 and Table 2).

In a double-blind pilot study, Sotirchos et al. demonstrated the dose-dependent effect of vitamin D on the serum level of IL-17 in pwMS [45]. In particular, they analyzed the serum level of IL-17 of 40 RR pwMS who were randomized to receive 10,400 IU (in 19 pwMS) or 800 IU (in 21 pwMS) of cholecalciferol daily for 6 months. In the group treated with a high dose of vitamin D, a reduction in serum IL-17+ CD4+ T cells and effector memory CD4+ T cells and an increase in central memory CD4+ T cells and naive CD4+ T cells were observed. These effects were not demonstrated in the group treated with a low dose of vitamin D. In terms of safety, the adverse events were minor, without differences between the two groups. The authors concluded that a high dosage (10,400 IU daily) of cholecalciferol supplementation had not only an immunomodulatory effect but also a safe and well-tolerated profile [42]. Hashemi et al. also confirmed the downregulation of IL-17 after vitamin D3 treatment (50,000 IU every week for 8 weeks) in a sample of 25 pwMS compared to 25 first-degree relatives of pwMS and 25 HCs [37].

Also, Golan et al. investigated the effect of different doses of vitamin D supplementation on serum IL-17 levels by randomizing 21 pwMS in a low-dose group (800 IU daily for 1 year) and 24 pwMS in a high-dose group (4370 IU daily for 1 year) [40]. Serum IL-17 levels were measured at baseline and after 3 months of vitamin D supplementation in 18 pwMS in the low-dose group and 20 pwMS in the high-dose group. Unexpectedly, a significant increase in serum IL-17 was found in the low-dose group, while non-homogenous trends in IL-17 levels were noted in the high-dose group. In the latter group, the serum IL-17 levels decreased in eight patients and increased in nine patients. In the other three patients, IL-17 levels were below the detection threshold, both at baseline and after 3 months of supplementation. Considering these results, the authors discussed the role of the possible confounding factors, which, however, could not be determined, although they suggested that one of these might have been represented by seasonal variations of immune cell subsets and cytokines [40].

Naghavi Gargari et al. confirmed a significant increase in the expression of IL-17A mRNA after treatment with 50,000 IU of vitamin D every week for 2 months, but only in the group of pwMS with an increase in serum vitamin D levels ≥ 20 nm compared to the baseline [44]. Also, Åivo et al. found a notable increase, although not significant, in serum levels of IL-17 in vitamin D-treated patients [42]. The authors discussed this result, suggesting that the increase in IL-17 might be attributed to elevated Transforming Growth Factor (TGF)-β activity, leading to the development of Th17 cells producing IL-17, interferon (IFN)-gamma, and IL-9 [42]. However, since pwMS were treated with IFN-β, it is not possible to exclude its possible effect on the cytokine responses to vitamin D.

Toghianifar et al. demonstrated that vitamin D supplementation (50,000 IU every five days for 12 weeks) was positively and significantly associated with the logarithm of serum IL-17 measurements in supplemented patients compared with the placebo group [52].

In contrast, either Smolders et al., Walawska-Hrycek et al., and Mrad et al. found no significant difference in the IL-17 serum levels [33,34,41] after vitamin D supplementation (20,000 IU/die for 12 weeks [34], 500–1000 UI/die for 12 months [26], or 10,000 IU daily for 12 weeks [41]). After vitamin D supplementation with 5000–10,000 IU/die for 24 weeks, O’Connell et al. confirmed no difference in IL-17+CD4+ T cells in people with CIS [43].

- **IL-6**

IL-6 is considered to have both a pro-inflammatory and potentially immune modulator role. IL-6 is known as a cytokine of acute phase reactions, but it also plays a role in the differentiation of B cells into plasma cells, the secretion of immunoglobulins, and in maintaining the balance between Th1 and Th2 effector functions [53].

In pwMS, increased levels of IL-6 have been found in plasma [54], CSF [55], and in brain acute and chronic active lesions [56]. Particularly, IL-6 is predominantly located within astrocytes at the sites of demyelination and immune activation [56]. In pwMS, peripheral blood B cells secrete more IL-6 compared with HCs [28,50], and enhanced signaling through the IL-6 receptor may induce a skewing of autoreactive T cells into a pathogenic Th17 phenotype [28].

The effect of vitamin D supplementation on the serum level of IL-6 in pwMS is still controversial. Hashemi et al. found a downregulation of IL-6 after vitamin D treatment (50,000 IU every week for 8 weeks) [37]. Conversely, Naghavi Gargari et al. found a significantly increased expression of mRNA after treatment with 50,000 IU of vitamin D every week for 2 months in the group with an Expanded Disability Status Scale (EDSS) of >2 and in the group of pwMS with serum vitamin D levels increased ≥20 nm compared to the baseline [44]. These data were not confirmed by another study published in the same year by Åivo et al., which did not find any change in the IL-6 serum levels after vitamin D treatment with 20,000 IU/week for 12 months [42] (see Table 1 and Table 2).

- **TGF-β1**

TGF-β is a versatile growth factor known to play a significant role in controlling essential processes during development and tissue repair, particularly in the central nervous system. It is a regulator of cell survival and differentiation, a mediator of cellular interactions, and it is implicated in the astrocyte differentiation and formation of astrocyte scars in response to brain injury [57], and TGF-β is able to promote neurogenesis. [58]

Moreover, TGF-β can regulate T cell expression in Th17-mediated autoimmune diseases [59], as well as ameliorate murine EAE by regulating NK-cell activity [60]. In particular, TGF-β1 levels in CSF samples of pwMS in the remission phase were significantly higher than in the active phase [61]. 

Already in 1998, Catorna et al. found that in the central nervous system of mice treated with 1,25-OH2 D3, the IL-4 and TGF-β1 transcripts were higher compared with the controls, and this increment was also correlated to a reduction in the total number of lymphocytes, demonstrating the potential anti-inflammatory effect of vitamin D mediated by an increase in the expression of these two cytokines [62]. Hawes et al. demonstrated that the expression of neurotrophin genes, brain-derived neurotrophic factor, and TGF-β1 was altered in mice with vitamin D deficiency [63].

These results were also confirmed in pwMS by measuring their TGF-β1 serum levels after vitamin D supplementation (see Table 1 and Table 2). Mahon et al. demonstrated that vitamin D supplementation (1000 IU vitamin D daily for 6 months) significantly increased the serum levels of TGF-β1 [46]. Hashemi et al. also confirmed the upregulation of TGF-β1 after vitamin D treatment (50,000 IU every week for 8 weeks) in a sample of 25 pwMS compared to 25 first-degree relatives of pwMS and 25 HCs (despite no statistical difference at the baseline in plasma concentrations of TGF-β1 among the three groups) [37]. Using latency-activated peptide (LAP) as a surrogate for bioactive TGF-β, Åivo et al. also demonstrated that the serum levels of TGF-β significantly increased after vitamin D (20,000 IU/week) treatment for 12 months [42]. This result on TGF-β was also confirmed by Walawska-Hrycek et al. (with a vitamin D supplementation dosage depending on the degree of deficiency, ranging from 500 to 1000 UI/die for 12 months) [33] and by Mosayebi et al. (after a monthly intramuscular injection of 300,000 IU of vitamin D for 6 months) [39].

On the other hand, Shirvani-Farsani et al. reported no change in TGF-β1 expression [30] but an increase in TGF-β2 mRNA expression [64] after vitamin D supplementation (50,000 IU every week via intramuscular injection for 8 weeks).

Muris et al. also reported no change in LAP of TGF-β secretion by T cells in pwMS treated with a high dose of vitamin D (7000 IU daily for 4 weeks, followed by 14,000 IU daily up to 48 weeks) [47].

Recently, Lozano-Ros et al. demonstrated that vitamin D could indirectly regulate the TGF-β pathway genes in RR pwMS [65]. In particular, they found an increased expression of the SMAD7 and ERK1 genes after supplementation with 0,266 mg/month of vitamin D for six months. These findings suggest a change in the TGF-β pathway due to vitamin D, carried out primarily through the non-SMAD pathway, linked to inhibition of the canonical SMAD pathway due to the overexpression of SMAD7 [65].

- **IFN-γ**

IFN-γ plays an important role in the innate and adaptive immune system. It acts by promoting the differentiation of Th1 cells and inhibiting the Th2 immune response, controlling the infiltration of macrophages and neutrophils into the CNS, and regulating the MHC class I and II protein expression and antigen presentation [66]. The pleiotropic effect of INF-γ on the immune system could explain its role in MS.

Initially, the induction of EAE in mice treated with IFN-γ [66] and the exacerbation of symptoms in pwMS treated with IFN-γ led to ascribing a pathological role to this cytokine in MS. Subsequent studies have demonstrated a protective role of IFN-γ in EAE. This two-faced role of IFN-γ in EAE and MS is still discussed, and it might be explained, at least in part, through its disease-stage-dependent dual action. In particular, IFN-γ could promote the pathogenesis of EAE, and its administration during the initiation phase of the disease leads to exacerbation. Conversely, IFN-γ might have a protective role during the effector phase [66].

As demonstrated by Galoppin et al., in pwMS 1,25-OH2 D3 reduces the production of IFN-γ by Th1 cells, probably for a direct effect on the VDR/RXR complex (bound to a silencer region in the human IFN-γ promoter) and for an indirect action on IL-12 production by dendritic cells and macrophages [67].

Mrad et al. confirmed a decreased IFN-γ production by stimulated T cells after 12 weeks of vitamin D supplementation (10.000 UI daily) in RR pwMS treated with IFN- β, which was associated with a significant increase in serum levels of vitamin D. The IFN-γ production was also negatively correlated with the baseline serum vitamin D [41].

Both Mahon et al. and Åivo et al. demonstrated that in RR pwMS, vitamin D supplementation (1000 IU daily for 6 months in the first study and 20,000 IU/week for 12 months in the second one) had no effect on IFN-γ [42,46]. This result was also confirmed by Golan et al. and Mosayebi et al. as well as by Smolders et al., who found no significant difference in the proportions of CD4+ T cells positive for IFN-γ after supplementation with 20,000 IU/die of vitamin D for 12 weeks [34,39,40]. However, when assessing the Th1/Th2 ratio, they found a decreased IFN-γ+/IL-4+ balance after supplementation, demonstrating a shift in the CD4+ T-cell cytokine profile from a pro- to an anti-inflammatory one, likely mediated by vitamin D. O’Connell et al. found no difference in IFN-γ + CD4+ T cells, even in people with CIS, after vitamin D supplementation with 5000–10,000 IU/die for 24 weeks [43].

In contrast, in RR pwMS, Walawska-Hrycek et al. found a significant increase in serum concentrations of IFN-γ after 12 months of vitamin D treatment (with the vitamin D supplementation dosage depending on the degree of deficiency, ranging from 500 to 1000 UI/die for 12 months) [33].

- **Other cytokines**

The effect on other cytokine levels was poorly investigated, and the principal results do not allow us to draw definitive conclusions.

Hashemi et al. observed the upregulation of IL-27 after vitamin D treatment (50,000 IU every week for 8 weeks) in a sample of 25 pwMS compared to 25 first-degree relatives of pwMS and 25 HCs [37].

Åivo et al. demonstrated that vitamin D supplementation (20,000 IU/week for 12 months) had no effect on the levels of IL-2, IL-9, IL-22, IL-13, IL-4, IL-5, IL-1β, and tumor necrosis factor (TNF)-α [42].

Mahon et al. demonstrated that, following vitamin D supplementation (1000 IU/day for 6 months), TNF-α and IL-13 were not different from the baseline levels, while the IL-2 mRNA levels decreased, although not significantly [46].

Also, Mrad et al. and Rolf et al. confirmed no significant effect on TNF-α serum levels after vitamin D supplementation (with 10,000 UI daily for 12 weeks or 7000 IU/day in the first 4 weeks, followed by 14,000 IU/day up to week 48) [41,48].

In the same year, Rolf et al. found no difference in the IL-2 receptor-alpha mRNA expression by PBMC of pwMS that received 14,000 IU/day of vitamin D for 48 weeks [68].

## 4. Discussion

Most studies reported that vitamin D supplementation leads to a significant increase in the serum concentrations of IL-10 and TGF-β1 but have not demonstrated an effect on IFN-γ (see Table 1). Instead, the results of studies on the serum levels of IL-17 and IL-6 in pwMS were contrasting despite being extensively investigated (see Table 1).

The contrasting results of some studies could be due to several factors.

The different dosages of vitamin D and treatment duration (see Table 1), but also different responses to vitamin D treatment and achieved serum levels after supplementation, might have influenced the outcomes of the different studies.

Indeed, the effect of vitamin D on IL-10 was mostly reported in studies that used a high dose of vitamin D supplementation. Åivo et al. and Golan et al. did not report any change in the levels of IL-10 when treating pwMS with a low dose of vitamin D despite a long treatment period (12 months) [40,42]. Åivo et al. discussed that the immunomodulatory effects were possible only with a higher dose of vitamin D supplementation [42]. Moreover, Shirvani-Farsani et al. demonstrated that there is a positive association between the serum concentrations of 25-OH D3 and the IL-10 levels [36]. Sotirchos et al. found an inverse correlation between the serum concentration of 25-OH D and IL-17 production: the absolute decrease in the percentage of IL-17+CD4+ T cells was only observed when the vitamin D serum level increased by >18 ng/mL compared to the baseline [45]. Although with contrasting results, Naghavi Gargari et al. also found that changes in the IL-6 and IL-17 levels were detected only with an increase in vitamin D serum levels ≥ 20 nm compared to the baseline [44]. The no-responder patients (∆vitamin D serum levels < 20, despite supplementation) showed no modulation of the cytokine profile [44].

De la Rubia Ortí demonstrated that the intake of vitamin D through daily nutrition was significantly and negatively related to IL-6 blood levels in a sample of 39 RR and SP pwMS, treated with glatiramer acetate or IFN- β: in particular, per each microgram (ug) of ingested vitamin D, IL-6 decreased 0.437 μg/mL [69].

Furthermore, the in vitro studies supported this dose effect of vitamin D: Niino et al. found that 1,25-OH2 D3 doses from 10−5 M to 10−9 M reduced the accumulation of IL-12/IL-23 (p40), IFN-γ, and TNF. On the other hand, the accumulation of IL-6 was not reduced by 1,25-OH2 D3 doses of 10−9 M [70].

Chen et al. showed that human primary B cells constitutively expressed low levels of VDR mRNA that were enhanced in the presence of 1,25-OH2D3 in a time-dependent manner [12].

Another factor to consider could be the baseline vitamin D serum level, which differed among the studies: Sotirchos et al. enrolled pwMS with values ranging between 20 and 50 ng/mL [45], Golan et al. [40] and Walawska-Hrycek et al. [33] with < 75 nmol/L, and Toghianifar et al. and Åivo et al. with <85 ng/mL [42,52]. The same Sotirchos et al. reported that the exclusion of pwMS with a vitamin D serum level of <20 ng/mL might have been a limitation because the immunomodulatory effect in response to cholecalciferol supplementation could be mostly evident in severe vitamin D deficiency [45].

The route of administration of vitamin D supplementation in most studies was oral, except for the studies conducted by Shirvani-Farsani et al. and Mosayebi et al. [36,39], which employed intramuscular injection. Shirvani-Farsani et al. [36] and Hashemi et al. [37] (both studies conducted in Iran) used the same dosage of vitamin D (50,000UI/week) for the same time period (8 weeks) but with different routes of administration: intramuscular for the first and oral for the second. Hashemi et al. demonstrated an increase in TGF-β1 serum levels [37], which was not found by Shirvani-Farsani et al. [36]. Due to the absence of a direct comparison, reaching conclusions about the optimal route of administration is not possible. Direct comparison studies between oral and intramuscular administration in non-MS individuals have demonstrated that the oral formulation seems to have a faster effect on vitamin D serum levels, whereas the intramuscular one seems more gradual. In particular, Cipriani et al. demonstrated that oral administration of vitamin D supplementation improved vitamin D serum levels at day 30, followed by a gradual decrease, but still remained significantly higher than the baseline up to day 90. Conversely, the intramuscular formulation slowly and constantly increased the vitamin D serum level, with the highest value observed at day 120 [71].

Despite the pharmacokinetic differences, both formulations have proven to be effective and safe. Therefore, it is not to prefer one route of administration over another, but rather, it is necessary to determine the optimal timing for the administration of vitamin D supplementation and evaluate its effect on the serum levels of vitamin D and cytokines. Further studies are necessary to compare the effect and to establish the right timing of administration and evaluation for the different formulations of vitamin D supplementation on cytokine serum levels in pwMS to identify the best strategy for obtaining a fast and sustained immunomodulatory response.

Treatment with DMTs could also influence the effect of vitamin D supplementation. The studies differed in the types of ongoing DMTs: Sotirchos et al. enrolled pwMS treated with IFN-β, glatiramer acetate, Natalizumab, and Fingolimod [45]; Walawska-Hrycek et al. enrolled those with IFN-β, glatiramer acetate, Natalizumab, Fingolimod, and Ocrelizumab [33]; and Golan et al. [40], Smolders et al. [34], and Åivo et al. [42] enrolled those only with IFN-β (1a or 1b). Therefore, IFN-β was the most commonly used DMT, and it is known that IFN-β treatment reduces IL-17 production via CD4+ T cells [11] and increases the circulating levels of IL-6, IL-10, and IFN-γ [72]. Therefore, treatment with IFN-β and vitamin D might have had a synergistic immunoregulatory effect [73]. Data on other DMTs are limited. However, it is possible to hypothesize a synergistic effect for other DMTs as well. In a murine model, the combination of vitamin D and Siponimod modulated the expression levels of microglia phenotype genes at the early and late remyelination stages [74]. In pwMS treated with Ocrelizumab, vitamin D serum levels of < 30 ng/mL were associated with a higher likelihood of early B cell reappearance at the six-month follow-up [18]. Vitamin D supplementation (associated with a significant increase in serum vitamin D concentrations) reduced the relapse rate in pwMS treated with Natalizumab [75].

Further studies categorizing pwMS treated with different DMTs are necessary to clarify the influence of other DMTs on the variation in serum levels of cytokines mediated by vitamin D supplementation and also to hypothesize a possible combination therapy.

However, serum cytokine levels also depend on other factors, primarily the disease phase (active vs non-active). Only in some studies are the MS phenotype and the relapse-free interval specified, and even when specified, there were differences among studies. For instance, Walawska-Hrycek et al. considered a relapse-free period of at least 6 months [33], Smolders et al. of at least 6 weeks [34], and Muris et al. [47], Toghianifar et al. [52], and Sotichors et al. [45] of at least 30 days. Furthermore, after enrollment, Golan et al. [40], Smolders et al. [34], and Sotichors et al. [45] reported three relapses (in one MS patient), one relapse, and two relapses, respectively. These data are highly relevant because the levels of cytokines change in the different disease phases: i.e., IL-17 and IL-6 levels increase in active MS lesions and during relapse [51,76]. Therefore, it cannot be excluded that recent or concurrent disease activity (either clinical or radiological) might have influenced the results of some studies. It would be interesting to categorize patients experiencing relapses and those without relapses during the study and the pre-enrolment period into two groups; such a categorization would allow us to investigate either the pure effect of vitamin D on the cytokine levels independently of relapses or, in case of relapse, occurrence.

Furthermore, the methodological differences in the processing of cells and the studied cells might have contributed to different outcomes. The different methods for each study are reported in Table 3.

The enzyme-linked immunosorbent assay (ELISA), polymerase chain reaction (PCR), and flow cytometry were the most used techniques (see Table 3). PCR measures the cytokine mRNA transcript, and ELISA detects the secreted cytokines at the protein level [77]. Flow cytometry enables the simultaneous identification of multiple cytokines at the individual cell level with a high throughput capability [77]. However, it involves several steps that could influence the result: the analysis time, the chosen stimulator reagent or protein transport inhibitor, and the cytokine to be detected.

Therefore, in choosing the method, it is necessary to consider several factors.

For example, the secretion of some cytokines is regulated at the translational or post-translational level (such as IL-4, IL-10, IL-1, and IL-8); therefore, the mRNA transcript does not always accurately reflect the circulating protein levels [77]. For instance, Favre et al., comparing PCR and ELISA, demonstrated that the level of TNF-α RNA detected by PCR did not correspond to the secreted level of TNF-α detected by ELISA. The same was demonstrated for IFN-γ in vivo (although the IFN-γ levels in vitro were the same for the PCR and ELISA) [78].

Rolf et al. compared distinct methods for assessing cytokine production by CD4+ T-cell-enriched CD3+CD8− lymphocytes in the context of vitamin D supplementation in pwMS and concluded that, especially in situations involving small sample sizes, the specific assay used to analyze the T cell cytokine profile is likely to be a significant factor affecting the reproducibility of study outcomes [79]. In particular, they demonstrated that stimulating PBMC with anti-CD3 led to an increase in the proportions of cells expressing various cytokines (such as IFN-γ, IL-4, IL-10, IL-17A, and GM-CSF), as detected by intracellular flow cytometry, with the exception of TNF-α. A moderate to strong correlation was demonstrated between the proportions of CD3+CD8− T cells positive for each cytokine, both directly ex vivo and after 72 h of pre-stimulation with anti-CD3, except for IL-10. The cytokine release in supernatants after 72 h of stimulation with anti-CD3 demonstrated a moderate to strong positive correlation with the proportions of IFN-γ, IL-4, and GM-CSF-positive cells measured by intracellular flow cytometry, with the exception of IL-10, IL-17A, and TNF-α [79].

Furthermore, a protein transport inhibitor, such as monensin, known to improve the cytokine detection for IFN-γ and IL-4, seems to impair the detectable fraction of IL-10+ T cells, whereas it does not influence the detection of IL-17A-producing T cells [80]. A possible alternative for the simultaneous detection of IFN-γ, IL-4, IL-10, and IL-17A might be an incubation with monensin for a shorter time period, i.e., 2 h, improving the detection of IL-10-producing T cells [80]. Moreover, monocytes treated with another protein transport inhibitor, brefeldin A, after 8 h in culture without stimulation, produced a percentage of IL-6 and TNF-a higher than monensin-treated monocytes [81]. Among the stimulator reagents, phorbol-12-myristate-13-acetate (PMA) plus Ionomycin might be preferred for cytokine-producing T cells, whereas lipopolysaccharides (LPS) might be preferred for cytokine-producing monocytes [82].

Therefore, the method’s choice and the timing of cell processing depend on the specific aim of the investigation, as well as the quantity and type of researched cytokines.

Niino et al. explained that the results of studies on the vitamin D effect on cytokine levels (particularly IL-10) may also be due to the different cell types studied; indeed, although VDR is present in various types of immune cells, it is expressed more significantly in CD8+ and CD4+ lymphocytes than in the monocyte/macrophage lineage, likely having a different effect size on the different cell types [70].

The effect of vitamin D on the IL-10 and IL-17 levels was also studied in vitro by da Costa et al.: 1,25-OH2D3 amplified the IL-10 production and decreased IL-17 release in T cell cultures from RR pwMS during clinical remission. The upregulated IL-10 production was related to the elevated percentage of IL-10-secreting T cells (including both the FoxP3+ CD25+ CD127− CD4+ Treg and the IL-10+ FoxP3− CD4+ Treg) [83].

In the murine model of EAE, the control of IL-10 levels by vitamin D was not merely significant but indispensable. According to Spach et al., mice with a defect in the IL-10-IL-10R pathway failed to show the inhibitory effects of vitamin D on EAE. This emphasizes the crucial role of the IL-10-IL-10R pathway in the anti-inflammatory and neuroprotective functions of vitamin D [84].

Lastly, it is important to note that some studies [36,40,44] lack a placebo-control group.

Therefore, although an anti-inflammatory effect of vitamin D supplementation might be possible, it appears to be achievable only at adequate dosages and with an appropriate response to serum levels. Vitamin D supplements may be recommended mostly in pwMS because of having lower levels of vitamin D compared to the general population [85]. Indeed, Esposito et al. reported that vitamin D was the most used food supplement (20.9%) among pwMS [86]. A Central and Eastern European Expert Consensus suggested vitamin D supplementation doses of 800 to 2000 IU/die for adults as prevention and higher dosages for treatment [87]. However, the dose used to modulate calcium and phosphate homeostasis may be different from the one used for immune system regulation, but the presently available data do not allow us to conclude on the appropriate dose and duration of treatment to obtain this response.

Except for the studies of Golan et al. and Walawska-Hrycek et al. [33,40], the administrated dose in most studies was higher than that used to prevent osteoporosis. Therefore, it is conceivable that the vitamin D supplementation doses for an immunomodulatory effect might exceed 800–2000 IU/die; however, establishing a standard dose is not possible. It should depend on the degree of deficiency and response to treatment (pwMS who do not respond to vitamin D may require higher dosages than responders). Additionally, the necessity of supplementation and vitamin D serum levels depends on various factors, such as sun exposition, age, BMI, and individual sensitivity to vitamin D supplements due to genetic, epigenetic, or nutritional factors, as well as comorbidities or concomitant medications.

Focusing on adverse events, Smolders et al. found an increase in serum creatinine levels during vitamin D3 supplementation, which was stable throughout all studies [34]. Åivo et al., Sotirchos et al., and Smolders et al. found no hypercalcemia, hypercalciuria, and no change in liver enzyme tests, demonstrating that a high dose of vitamin D supplementation had a safe and well-tolerated profile [34,42,45].

Therefore, the effect on cytokine levels revealed a potential anti-inflammatory effect of vitamin D supplementation. While some studies reported no change in the EDSS and relapse rates, other ones provided evidence of a clinical benefit, supporting the anti-inflammatory effect of vitamin D [39,40]. Naghavi Gargari et al. observed a reduction in EDSS scores (from a mean of 2.21 ± 1.03 to 1.96 ± 1.05; *p* = 0.002) following vitamin D supplementation [44]. Furthermore, da Costa et al. found that 1,25-OH2D3 supplementation increased the percentage of IL-17-producing (CD4+ and CD8+) T cells co-expressing IL-10, and this higher proportion of IL-10-producing Th17 cells correlated with lower EDSS scores [83].

Walawska-Hrycek et al. found that, after one year of Vitamin D supplementation, no changes in EDSS occurred; however, the correlation between EDSS and vitamin D levels was not assessed [33].

The clinical and radiological implications of the effect of vitamin D supplementation on the cytokine profile in pwMS should be further investigated, as they are missing in several studies [34,37,45,47].

The effects of reducing the relapse rate, disease progression, the rate of new/enlarging T2 lesions, number of T1-enhancing lesions, and brain volume loss have been reported in several studies [15,16,17,18]; however, integrating these data with variation of cytokine profiles due to vitamin D supplementation could be useful to identify, and possibly predict, the clinical and radiological effect.

## 5. Conclusions

Cytokines play a crucial role in cell signaling and, consequently, in the development, differentiation, and regulation of immune cells. Dysregulation of the cytokine profile was found in pwMS, compared with HCs; in particular, peripheral blood B cells secrete less IL-10 and more IL-6, and T cells present resistance to IL-10-mediated immunosuppression. Furthermore, the presence of Th17 cells and high levels of IL-17 were detected in the active phase of the disease, compared to the remission phase or other non-inflammatory neurological diseases. The immunomodulatory effect of vitamin D may suggest a possible favorable role of vitamin D supplementation in pwMS.

Most studies reported that in pwMS, vitamin D supplementation leads to a significant increase in the serum concentrations of IL-10 and TGF-β1, pointing to a potential effect of vitamin D toward an anti-inflammatory cytokine profile. Thus far, the clinical evidence demonstrates a reduction in the EDSS scores after vitamin D supplementation and an inverse correlation between the IL-10 levels and EDSS scores.

However, studies on vitamin D supplementation in pwMS, integrating laboratory, clinical, and radiological parameters are still limited.

Furthermore, in pwMS, no effect of vitamin D supplementation on IFN-γ was demonstrated, and despite extensive investigation, the results of the studies on serum levels of IL-17 and IL-6 in pwMS were conflicting, likely due to methodological factors.

Further high-quality trials on vitamin D’s supplementation effect, including larger cohorts of pwMS and control groups, are eagerly awaited. Adequate treatment duration, the best route and frequency of administration, vitamin D dosage supplementation, response patterns to vitamin D treatment, target serum levels for anti-inflammatory effects, evaluation of clinical and radiological outcomes, cytokine analysis methods, and relevant cell types still need to be identified. This should be done in relation to the MS phenotype, disease duration, and concomitant DMT.

## Figures and Tables

**Table 1:** Effect of vitamin D supplementation on cytokine levels.

Cytokine	Study	Vitamin D Treatment			Results	Note
		Dosage	Duration	Route of Administration		
IL-10	Walawska-Hrycek, 2021 [33]	500 to 1000 UI/die	12 months	oral	↑	
	Smolders, 2010 [34]	20,000 IU/die	12 weeks	oral	↑	
	Ashtari, 2015 [35]	50,000 IU/5 days	3 months	oral	↑	
	Shirvani-Farsani, 2015 [36]	50,000 IU/week	8 weeks	i.m.	↑	
	Hashemi, 2018 [37]	50,000 IU/week	8 weeks	oral	↑	
	Hashemi, 2020 [38]	50,000 IU/week	8 weeks	oral	↑	
	Mosayebi, 2011 [39]	300,000 IU every month	6 months	i.m.	↑	
	Golan, 2013 [40]	800 IU daily vs 800 IU daily + 75,000 UI every 3 weeks	12 months	oral	↔	
	Mrad, 2017 [41]	10,000 IU/die	12 weeks	oral	↔	
	Åivo, 2015 [42]	20,000 IU/week	12 months	oral	↔	
IL-17	Walawska-Hrycek, 2021 [33]	500 to 1000 UI/die	12 months	oral	↔	
	Smolders, 2008 [34]	20,000 IU/die	12 weeks	oral	↔	
	O’Connell, 2017 [43]	5000–10,000 IU/die	24 weeks	oral	↔	
	Mrad, 2017 [41]	10,000 IU/die for	12 weeks	oral	↔	
	Naghavi Gargari, 2015 [44]	50,000 IU/week	2 months	oral	↑	
	Golan, 2013 [40]	800 IU/die vs 800 IU/die + 75,000 UI every 3 weeks	12 months	oral	↑	Only in the low-dose group after 3 months
	Åivo, 2015 [42]	20,000 IU/week	12 months	oral	↑ *	
	Hashemi, 2018 [37]	50,000 IU/week	8 weeks	oral	↓	
	Hashemi, 2020 [38]	50,000 IU/week	8 weeks	oral	↓	
	Sotirchos, 2016 [45]	10,400 IU or 800 IU daily	6 months	n.s.	↓	Only in the group treated with high-dose
IL-6	Naghavi Gargari, 2015 [44]	50,000 IU/ week	2 months	oral	↑	
	Åivo, 2015 [42]	20,000 IU/week	12 months	oral	↔	
	Hashemi, 2018 [37]	50,000 IU/week	8 weeks	oral	↓	
	Hashemi, 2020 [38]	50,000 IU/week	8 weeks	oral	↓	
TGF-β1	Hashemi, 2020 [38]	50,000 IU/week	8 weeks	oral	↑	
	Mahon, 2003 [46]	1000 IU/die	6 months	oral	↑	
	Walawska-Hrycek, 2021 [33]	500 to 1000 UI/die	12 months	oral	↑	
	Mosayebi, 2011 [39]	300,000 IU every month	6 months	i.m.	↑	
	Åivo, 2015 [42]	20,000 IU/week	12 months	oral	↑	
	Muris, 2016 [47]	7000 IU/die for 4 weeks, followed by 14,000 IU/die	48 weeks	oral	↔	
	Shirvani-Farsani, 2015 [36]	50,000 IU/week	8 weeks	i.m.	↔	
IFN-γ	Mahon, 2003 [46]	1000 IU/die	6 months	oral	↔	
	Åivo, 2015 [42]	20,000 IU/week	12 months	oral	↔	
	Mosayebi, 2011 [39]	300,000 IU every month	6 months	i.m.	↔	
	O’Connell, 2017 [43]	5000–10,000 IU/die	24 weeks	oral	↔	
	Golan, 2013 [40]	800 IU/die vs. 800 IU/die + 75,000 UI every 3 weeks	12 months	oral	↔	
	Mrad, 2017 [41]	10,000 IU/die	12 weeks	oral	↓	
	Walawska-Hrycek, 2021 [33]	500 to 1000 UI/die	12 months	oral	↑	

Legend: IL: interleukin; TGF: transforming growth factor; IFN: interferon; n.s.: not specified route of administration; i.m.: intramuscular injection; ↑: increase in cytokines; ↓: reduction in cytokines; ↔: no effect on cytokines; *: not significant.

**Table 2:** People with multiple sclerosis characteristics in different studies.

Study	Country	Sample Size			Sex (F/M)	Age (Years)	Disease Duration	EDSS at Baseline	Phenotype of MS	ARR
Walawska-Hrycek, 2021 [33]	Poland	44			33/11	38.4 ^mn^	5.6 ys ^mn^	1.5 ^mdn^	RR	n.i.
Smolders, 2008 [34]	The Netherlands	15			8/7	35.15 ^mdn^	3.47 ys ^mdn^	2.0 ^mdn^	RR	1.0 ^mdn^
Ashtari, 2015 [35]	Iran	DG	44		35/9	31.5 ^mn^	4.10 ys ^mn^	1.70 ^mn^	RR	0.75 ^mn^
		PlG	45		40/5	34.6 ^mn^	4.46 ys ^mn^	2.09 ^mn^		0.83 ^mn^
Shirvani-Farsani, 2015 [36]	Iran	DG	32		27/5	29.78 (F) ^mn^ 37.4 (M) ^mn^	6.40 ys ^mn^	2.12 ^mn^	RR	n.i.
		CG	32		23/9	28.6 (F) ^mn^ 27.57 (M) ^mn^	-	-	-	
Hashemi, 2018 [37]	Iran	MSP	25		21/4	32.6 ^mn^	8.1 ys ^mn^	n.i	n.i	n.i
		RP	25		17/8	27.4 ^mn^	-			
		CG	25		20/5	31.7 ^mn^	-			
Hashemi, 2020 [38]	Iran	MSP	25		21/4	32.6 ^mn^	8.1 ys ^mn^	n.i	n.i	n.i
		RP	25		17/8	27.4 ^mn^	-			
		CG	25		20/5	31.7 ^mn^	-			
Mosayebi, 2011 [39]	Iran	DG	26		17/9	37 ^mn^	4.15 ys ^mn^	2.1 ^mn^	RR	n.i. #
		CG	33		25/8	35 ^mn^	6.4 ys ^mn^	2.5 ^mn^	-	
Golan, 2013 [40]	Israel	LDG	21		13/8	44.7 ^mn^	9.3 ys ^mn^	3.6 ^mn^	RR	0.38 ^mn^
		HDG	24		19/5	43.1 ^mn^	6 ys ^mn^	2.9 ^mn^	RR	0.28 ^mn^
Mrad, 2017 [41]	Lebanon	46			24/22	34.6 ^mn^	3.8 ^mn^	1	RR	n.i.
Åivo, 2015 [42]	Finland	DG	30		18/12	38 ^mdn^	3 ys ^mdn^	2.0 ^mdn^	RR	0.5 ^mdn^
		PlG	29		19/10	35 ^mdn^	2 ys ^mdn^	1.5 ^mdn^	RR	0.5 ^mdn^
Naghavi Gargari, 2015 [44]	Iran	32			26/6	30.68 ^mn^	5.65 ys ^mn^	2.21 ^mn^	RR	n.i.
Sotirchos, 2016 [45]	USA	HDG	19		14/5	41.3 ^mn^	8.2 ys ^mn^	3 ^mdn^	RR	n.i.
		LDG	21		14/7	38.8 ^mn^	7.8 ys ^mn^	2 ^mdn^	RR	
Mahon, 2003 [46]	USA	39			n.i.	n.i.	n.i.	n.i.	n.i./	n.i.
Muris, 2016 [47]	The Netherlands	DG	30		21/9	37.7	7.3 mts	≤3.5:22 4–5.5:1	RR	1.26 *
		PlG	23		14/9	37.2	7.3 mts	≤3.5:28 4–5.5:2	RR	1.67 *
O’Connell, 2017 [43]	Ireland	CIS	HDG	12	8/4	37.2 ^mn^	-	0.9	CIS	
			LDG	10	5/5	32.7 ^mn^		0.9		
			PlG	7	6/1	34.3 ^mn^		0.4		
		Ctr	HDG	13	6/7	30.5 ^mn^			-	
			LDG	13	10/3	30.3 ^mn^				
			PlG	12	10/2	29.1 ^mn^				

Legend: mdn: median; mn: mean; DG: group treated with vitamin D; Ctr: control group; PlG: placebo group; LDG: low-dose group; HDG: high-dose group; MSP: multiple sclerosis participant; RP: first-degree relative participant; RR: relapsing-remitting MS; n.i.: not indicated; CIS: clinically isolated syndrome; ys: years; mts: months; *: number of attacks during the past 2 years at baseline; #: in the inclusion criteria: at least 1 relapse in the previous 12 months.

**Table 3:** Methods for each study.

Study	Method
Walawska-Hrycek, 2021 [33]	ELISA
Smolders, 2008 [34]	antiCD3 iFACS (PMA + iono/mone)
Ashtari, 2015 [35]	
Shirvani-Farsani, 2015 [36]	PCR
Hashemi, 2018 [37]	PCR
Hashemi, 2020 [38]	PCR
Mosayebi, 2011 [39]	ELISA
Golan, 2013 [40]	ELISA
Mrad, 2017 [41]	antiCD3/antiCD28 CBA iFACs (PMA + iono/B_A_)
Åivo, 2015 [42]	FBA
O’Connell, 2017 [43]	antiCD3 ELISA iFACs (PMA + iono/B_A_)
Naghavi Gargari, 2015 [44]	PCR
Sotirchos, 2016 [45]	antiCD3/antiCD28 iFACs (PMA + iono/B_A_-mone)
Manhor, 2003 [46]	PCR
Muris, 2016 [47]	antiCD3 iFACs -PMA + iono/BA -> B-cell -PMA/mone -> T cell (no mone for IL-10) Multiple immunoassay

Legend: ELISA: enzyme-linked immunosorbent assay; PCR: polymerase chain reaction; iFACs: intracellular flow cytometry; antiCD3: stimulation with antibody anti-CD3; CD28: stimulation with antibody anti-CD28; mone: monensin; BA: brefeldin A; PMA: phorbol-12-myristate-13-acetate; iono: Ionomycin; IL: interleukin.

## Data Availability

Not applicable.
